# Relationship of Racial Composition and Cancer Risks from Air Toxics Exposure in Memphis, Tennessee, U.S.A.

**DOI:** 10.3390/ijerph110807713

**Published:** 2014-07-31

**Authors:** Chunrong Jia, Wesley James, Satish Kedia

**Affiliations:** 1 School of Public Health, University of Memphis, Memphis, TN 38152, USA; E-Mail: skkedia@memphis.edu; 2 Department of Sociology, University of Memphis, Memphis, TN 38152, USA; E-Mail: wljames1@memphis.edu

**Keywords:** air toxics, cancer risks, racial disparity, NATA

## Abstract

African Americans in the U.S. often live in poverty and segregated urban neighborhoods, many of which have dense industrial facilities resulting in high exposure to harmful air toxics. This study aims to explore the relationship between racial composition and cancer risks from air toxics exposure in Memphis/Shelby County, Tennessee, U.S.A. Air toxics data were obtained from 2005 National Air Toxics Assessment (NATA), and the demographic data, including racial composition, were extracted from the 2000 United States Census. The association was examined using multivariable geographically weighted regression (GWR) analysis. The risk difference between African American and White concentrated areas was defined as the absolute disparity, and the percent difference as the relative disparity. GWR analyses show that cancer risks increase with respect to increasing percent of African Americans at the census tract level. Individuals in African American concentrated tracts bear 6% more cancer risk burden than in White concentrated tracts. The distribution of major roads causes the largest absolute disparity and the distribution of industrial facilities causes the largest relative disparity. Effective strategies for reduction in environmental disparity should especially target sources of large absolute disparities.

## 1. Introduction

**Figure 1:**
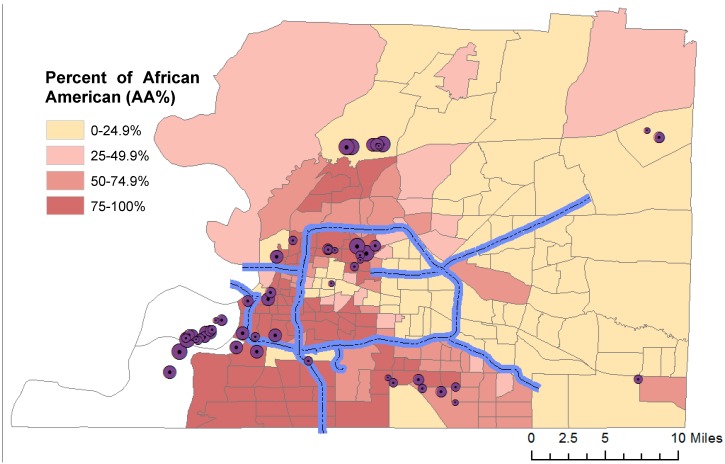
Map of Memphis/Shelby County, Tennessee. Dots show the major point emission sources reported in 2005 TRI. Highlighted road sections are high traffic roads with average annual daily traffic (AADT) > 50,000. Blank tracts have no permanent housing.

African Americans in the U.S. are faced with significant environmental and health disparities, especially in the Mid-South region. Shelby County, Tennessee, is a Mid-South metropolitan region with a population of 930,000, the largest in the state. The county seat Memphis has a population of 650,000, making it the most populous city in Tennessee. The county has a much higher concentration of African Americans (53%) compared to Tennessee (17%), and Memphis has an even higher minority residential concentration of 63% [1]. Within the county, African Americans comprise the vast majority of the western (downtown Memphis) and southern parts of the city of Memphis, but are also distributed across northern Memphis in large numbers. Significantly fewer African Americans reside in eastern Shelby County tracts, the location of Memphis’ largest suburbs (Figure 1). In general, African Americans have higher poverty rates and poorer health conditions [2]. Certain factors are known to contribute to poor health conditions among African Americans, such as low access to preventative care, lack of health insurance or underinsurance, and unhealthy lifestyle [3]. In addition to these factors, environmental pollution in residential areas has been recognized as a major health risk for minorities [4]. Many of the major minority health issues in Memphis are environmentally related [5], including but not limited to poor birth outcomes [6], high prevalence of asthma [7], cardiovascular diseases [8], cancers, and general mortality [9].

Memphis is a hub for many major industries such as transportation carriers, a petroleum refinery, petrochemical storage and transfer facilities, waste disposal facilities, and a power plant, all of which emit air toxics in the vicinity. The Environmental Protection Agency’s (EPA) Toxic Release Inventory (TRI) database indicates that major air toxics in Memphis include many carcinogens, e.g., benzene, 1,3-butadiene, chloroform, and acrylonitrile [10]. Like most urban locations in the U.S. [11], the major industrial facilities in Memphis are primarily clustered in or close to minority neighborhoods, including South and North Memphis, Millington, and Presidents Island. The major roads are also located in minority concentrated and/or low-income areas (Figure 1). Minorities, often of low socioeconomic status (SES), continue to be at higher risks of environmentally related diseases, e.g., pulmonary, cardiovascular, and malignant diseases. Yet the differential exposure to environmental pollutants has received little attention by either government entities or civic organizations in this region.

In the U.S. urban context, high minority concentrations and low SES are closely associated, which lends itself to disproportionate burden of air pollution exposures [4]. A general hypothesis is that emission sources are often concentrated in these disadvantaged areas and as a result, minorities have higher levels of exposure to air toxics and the associated health risks. This study examines how the uneven spatial distribution of air toxics sources causes disproportionate environmental exposure among African American concentrated tracts in Memphis/Shelby County. For convenience, we use the county seat Memphis for the description, but all the analyses are based on data in Shelby County. The Memphis area represents a typical metropolitan setting that consists of industrial, downtown, suburban, and rural areas and covers various levels of industrialization and urbanization in different localities. To our knowledge, this is the first study to assess environmental health disparities in the Mid-south region. The results of this study will further our understanding of existence, scale, and location of disparities in air pollution exposure and inform policy makers to reduce harmful emissions in these minority concentrated areas of this region and across the country.

## 2. Methods

### 2.1. Data Sources

Racial composition and socioeconomic status (SES). Census-tract level racial composition and SES data were obtained from the Census 2000, Summary File 3 [12]. The following variables were extracted: percent of the population that is African American (shortened as “AA%”), median household income in 1000s, percent of the population below the poverty level, percent of female headed households with children, percent of the population with less than a high school education, total population, population density (1000 person /mile^2^), and percent of the population aged 65 and older. Based on AA%, tracts were categorized as African American concentrated (AA% ≥ 75%, shortened as AA area), mixed race (25% ≤ AA% < 75%, shortened as mixed area), and White concentrated areas (AA% < 25 %, shortened as White area) [13].

Cancer risk. Estimates of cancer risks from exposure to air toxics were obtained from 2005 National-Scale Air Toxics Assessment (NATA). EPA has been operating the NATA program since 1999 to provide estimates of concentrations, exposures, and risks of air toxics. The 2005 NATA database, released in 2011, is the latest available data. NATA models ambient concentrations using major source emissions databases, Toxics Release Inventory (TRI), National Emissions Inventory (NEI), and meteorological data. According to a recent report that made model-to-monitor comparisons for over 800 sites, the modeled concentrations in 2005 NATA generally agree with monitoring results [14]. Population exposures to ambient toxics are then modeled using ambient concentrations and time-activity patterns. Finally, lifetime excess cancer risks are estimated in a linear no-threshold model: Risk = Exposure × IUR (inhalation unit risk). A risk is expressed as a probability, e.g., 10^−4^ means that 1 person out of 10,000 is expected to contract cancer due to the life-time exposure. The EPA considers a risk of concern if it exceeds the benchmark of 1 in one million. NATA evaluates cancer risks of 80 chemicals that have cancer potencies [15] and the cumulative (total) risk, which is defined as the sum of all these carcinogens assuming the additivity of risks. The use of cancer risks to examine environmental health disparities has two advantages: first, cancer risk directly reflects adverse health conditions rather than an exposure level that is hard to interpret; second, the risk normalizes the exposure by the chemical’s toxicity, so that the health effects from different toxics with different concentrations are comparable. As NATA provides cancer risk estimates at the census tract level, many case studies have used NATA estimates to examine environmental disparities at local, state, and regional levels [16,17,18]. Cancer estimates at the census tract level were extracted for Shelby County, Tennessee from the 2005 NATA database [19]. Spatial information of the census tracts was obtained as shapefiles from US Census Bureau’s 2000 TIGER/Line Shapefiles [20].

### 2.2. Groupings of Racial, SES, and Population Variables

Representative variables were identified using principal component analysis, as race and SES are often associated. By selecting variables with loadings of ≤−0.5 or ≥0.5, two factors were obtained: Group 1 variables reflect SES and racial composition, and Group 2 variables indicate population characteristics (Table A1). This analysis indicates that race and SES variables are highly correlated, as reported previously [16]. To avoid the co-linearity issue, we used AA% and population density for the following multivariable analysis.

### 2.3. Geospatially Weighted Regression (GWR) Analysis

We explore how tract-level cancer risks, due to exposure to air toxics, are associated with racial composition using geospatially weighted regression (GWR) analysis. This study focuses on the cumulative risk that reflects the exposure burden from all the carcinogenic chemicals. As EPA targets source control to reduce risks, the analysis also used partial cumulative risks contributable to point, nonpoint, on-road, and non-road sources. Point and on-road sources are industrial facilities and roads described in proximity analysis; and nonpoint and non-road sources are area sources. Secondary sources are those from the atmospheric transformation of other chemicals. Background concentrations are “the contributions to outdoor air toxics concentrations resulting from natural sources, persistence in the environment of past years’ emissions, and long-range transport from distant sources” [21]. Secondary sources and background concentrations are not geographically variable (coefficients of variance of 212 tracts = 4.3% and 1.6%, respectively) within this county, and thus they were excluded from the GWR analysis. The data pertaining to cancer risk, racial composition, and location were linked by the census tract number. Two census tracts without permanent housing were excluded from the analysis.

In the GWR model, risk (unit: 1 in one million) is the dependent variable and AA% is the explanatory variable. Population density (unit: 1000 person/km^2^) is also included as a covariate, as densely populated or urbanized areas have more air pollution sources that impacts residents’ health. This model indicates the association rather than the causality between dependent and independent variables. The frequency distributions of risks showed right-skewed distributions, and thus risk values were log-transformed. Spatial autocorrelation was detected by Moran’s I tests for variables used in models. Here, we used a spatial error model to account for spatial autocorrelation [22]:

Ln(Risk) = β_0_ + β_1_ AA% + β_2_ ln(Popden) + λWu + e (1)

where λ represents the coefficient for spatially autocorrelated errors (spatial autoregressive coefficient), W is the spatial weights matrix based on tract centroids, e represents the random error term in the ordinary linear square (OLS) model, and u is the spatially independent error term. The parameters of this spatial error model are estimated using the maximum likelihood method. The spatial error model has been demonstrated to be appropriate for census tract data [22]. It overcomes the issues otherwise brought by the ordinary least square (OLS) regressions without considering spatial dependence, such as biased/inconsistent estimates of regression coefficients and exaggerated R^2^ [22]. The distance-based spatial weights were constructed using a threshold distance of 2-km. The critical distance of 2-km was selected as air pollution and the associated health risks are negligible beyond this distance [23]. The GWR regression models were then run for cumulative risk and risks from point, non-point, on-road, and non-road sources in OpenGeoDa (Version 1.4.6, GeoDa Center, Tempe, AZ, USA).

When AA% in Model (1) is categorized, we can compare cancer risks among AA, mixed, and White areas. We then defined the risk difference between two areas as the absolute disparity, and the percent difference as the relative disparity. We are particularly interested in the disparities between AA and White areas.

## 3. Results

### 3.1. Higher Cancer Risks in Memphis Populations

The Memphis population, in general, is at high cancer risk due to exposure to multiple air toxics (Table 1). At the county level, the cumulative cancer risk was 55 × 10^−6^, meaning that 55 persons out of 1 million are expected to contract cancers due to the exposure to all carcinogenic air toxics over their lifetime. Given the population size of around 1 million in the study area, this represents approximately 55 additional number of cancer cases resulting from air pollution. This cancer risk level is higher than the 95th percentile risk levels both for Tennessee and the nation (Table 1), indicating that Memphis residents are at the high end of air toxics exposure in the U.S. The three largest contributors are secondary sources, background concentrations, and on-road emissions, contributing 53%, 20%, and 13% of the cumulative risk, respectively. As secondary pollutants are formed due to the atmospheric reactions of primary pollutants, the high level of secondary sources actually reflect high quantities of point, nonpoint, on-road, and non-road emissions.

Seven specific air toxics have cancer risks that exceed 1 × 10^−6^, the benchmark level of concern [24]. The risks of these compounds ranged from 1.2 to 28.7 × 10^−6^ in Memphis, all higher than the 95th percentiles of the state and/or national levels. Together they contributed 89% of the total risk, and they were the major “risk contributors” in this area. In particular, formaldehyde and benzene contributed 52% and 15% of the cumulative risk, and could be considered “risk drivers” that posed the highest cancer risks. In the atmosphere, formaldehyde is contributed primarily by photochemical oxidation of reactive organic gases with ozone and nitrogen oxides [25], and its risk is almost entirely from secondary sources. Inorganic arsenic and carbon tetrachloride were ubiquitous pollutants contributable to background concentrations.

**Table 1:** Cancer risks (unit: 10^−6^) of populations in the U.S., the State of Tennessee, and Memphis/Shelby County.

Area	The U.S.				Tennessee				Memphis
Sample size	(*n* = 3222)				(*n* = 95)				(*n* = 1)
Statistics	Mean	Median	95th		Mean	Median	95th		
**By source**									
Point	0.54	0.17	2.09		0.73	0.24	3.64		1.12
Nonpoint	2.69	1.72	9.07		1.99	1.68	4.25		3.56
On-road	1.73	0.75	7.05		1.59	0.87	5.38		6.86
Non-road	0.69	0.35	2.45		0.54	0.37	1.55		3.23
Background	7.16	6.77	11.49		7.64	7.52	11.47		10.71
Secondary	17.34	16.56	28.86		23.18	23.39	27.27		29.19
Cumulative	30.16	28.96	50.97		35.67	34.49	50.39		54.68
**By compound**									
Formaldehyde	15.88	15.14	26.64		20.31	20.29	24.54		28.69
Benzene	3.21	2.82	7.80		3.31	2.98	7.90		8.18
Acetaldehyde	2.78	2.66	4.59		3.78	3.80	4.40		4.73
Carbon tetrachloride	2.85	2.85	2.87		2.85	2.86	2.88		2.86
1,3-Butadiene	0.61	0.45	1.80		0.54	0.45	1.38		1.68
Arsenic	0.73	0.39	2.04		0.54	0.40	1.56		1.56
Naphthalene	0.62	0.38	2.06		0.49	0.41	1.08		1.21

### 3.2. Significant Racial Disparities in Cancer Risks

The GWR models confirm that cancer risks are highly associated with pockets of minority concentrations in Memphis (Table 2). The high R^2^ of 0.68 for the cumulative risk model indicates that race and population density can easily explain the distribution of risks. All the models accounted for spatial autocorrelation, as indicated by Moran’s I tests, except for the risk from non-road sources. Considering the marginal p-value (between 0.01 and 0.05), the model results are acceptable.

**Figure 2:**
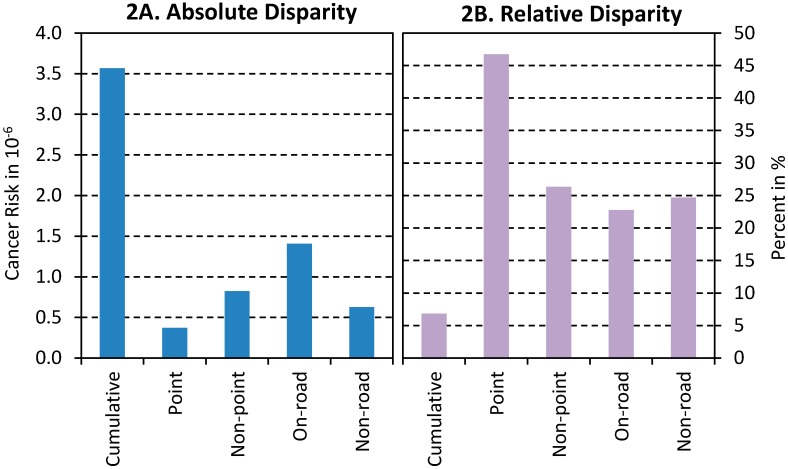
Absolute and relative disparities between African American and White concentrated areas (White as the reference).

In Table 2, the coefficient for “AA%” means increased risk in more African American concentrated tracts. As risk values were log-transformed, the coefficient of the cumulative risk (0.084) is interpreted as a 0.084% increase of risk per every percentage point increase of AA%. The disparities become substantial when racial composition is classified. Regarding the relative disparity, people in AA areas have 6% more risk than those in White areas. Using the average risk of 52.1 × 10^−6^ in White areas as the reference, AA areas have an extra risk of 3.6 × 10^−6^ (Figure 2A), equivalent to the sum of 1,3-butadiene and arsenic risks (Table 1). In other words, the absolute disparity between AA and White areas is significant, and it is equivalent to two major risk contributors. Absolute disparities of risks from point, nonpoint, on-road and non-road sources contributed 10%, 23%, 39%, and 18% to the total disparity of 3.6 × 10^−6^, respectively (Figure 2A); collectively, they contributed 90% of the total disparity.

**Table 2:** Association of cancer risk and race. Popden: population density (1000 person/km^2^) at the census tract level. Moran’s I: *p*-value of the Moran’s I test for model residuals.

Risk Sources	AA%	*p*-Value		Popden	*p*-Value		R^2^	Moran’s I
Point	0.512	<0.001		−0.012	0.370		0.73	0.232
Nonpoint	0.235	0.003		0.063	0.000		0.54	0.597
On-road	0.300	0.009		0.053	<0.001		0.64	0.178
Non-road	0.256	0.001		0.055	<0.001		0.75	0.024
Cumulative	0.084	0.001		0.013	<0.001		0.68	0.343

The relative disparity, expressed by percent difference, is more prominent if examining the risks from individual emission sources (Figure 2B). The coefficients for point, nonpoint, on-road and non-road sources mean 0.5%, 0.2%, 0.3%, and 0.3% increase every percentage point increase in AA%, respectively. These rates can be translated into relative disparities of 38%, 23%, 21% and 22%, respectively, between AA and White areas (Figure 2B). Obviously, the relative disparity of risks from point sources is the highest.

### 3.3. Higher Risks in More Densely Populated Areas

Cancer risks are higher, in general, in areas with higher population density. The coefficient of 0.013 means that an increase of 1000 person/km^2^ causes an increase of 13% in the cumulative risk. The coefficients are larger for risks from specific sources except for point sources, indicating that nonpoint, on-road and non-road sources are located in or close to more populated areas. The insignificant coefficient for risk from point sources, *i.e.*, industrial facilities, is caused by the large industrial facilities that are located on Presidents Island, which does not have residential areas, and in northern Shelby County, which is sparsely populated. Overall, air toxics pollution poses higher cancer risk in densely populated areas, which may result in higher health care costs in the impacted area.

## 4. Discussion

The analyses of the latest NATA data indicate that Memphis is at the high end of environmental exposure to air toxics in the U.S. Within this area, environmental disparity is significant, *i.e*., disproportionate exposure to air toxics occurs in African American concentrated census tracts. These findings are consistent with those obtained in other metropolitan cities and regions with high air pollution in the U.S., e.g., St. Louis, Missouri [26], Houston, Texas [18], California’s South Coast Air Basin [27] and Louisiana’s Cancer Alley region between Baton Rouge and New Orleans [16].

### 4.1. Risk Perception and Disparity Contributors

The perception of environmental risks depends on the nature of pollution sources. Industrial facilities, *i.e.*, the point sources, are always the areas of greatest concern for environmental justice efforts. The general public usually perceives industries to be the biggest culprit of environmental pollution, which is the case in many instances. The clustering of industries in Memphis has historically been reflective of inequities in power, racial segregation, and discriminatory practices in community planning and zoning, similar to many other U.S. urban areas [28]. There have been stories of communities fighting against the impacts of landfills, chemical plants, and other pollution-intensive industries in Memphis [29]. However, there are other equally or sometimes, more damaging sources of air toxics, especially in the U.S. urban context. Nonpoint, traffic, and non-road emissions sources are also called “people pollution” because much of it is the result of activities that people engage in their daily lives. Our analysis shows that relative disparity caused by distribution of point sources is the largest (38%), and the relative disparities caused by the other sources are smaller (21%–23%). Our measure of relative disparity reflects the public’s perception of environmental risks: people are more resistant to industries whose distribution shows high relative disparities, but perceive other pollution sources, whose distribution shows low relative disparities, more acceptable.

In contrast, the perceived disparity is not always reflective of the absolute disparity. Uneven distribution of point sources contributes the smallest portion (10%) of the total absolute disparity. This is mainly due to stricter control and less emission of industrial chemicals over a period of many years [30]. The on-road traffic emissions are the largest contributor (39%) to the total absolute disparity. However, this portion of disparity does not raise concern among people due to lack of awareness. In addition, low-income minorities do not have many options and end up living near major roads due to low housing costs.

In short, while there is a large relative disparity from point sources, this disparity is at such a low absolute level that eliminating it would do little to address the overall disparity in health risk from all pollutant sources. On the contrary, although there is a small relative disparity in health risk from on-road sources, the absolute difference is high enough that reducing this disparity would go a long way towards reducing the overall racial disparity in air toxics exposure. Effective environmental interventions should target the sources that create the largest absolute disparity, such as on-road sources.

### 4.2. Implications and Future Research

While previous studies are unclear about the role of racial segregation in environmental disparities in the Mid-south region [31], this study provides empirical evidence that spatial distribution of minorities are the driving predictors of environmental disparities, as reported in an earlier study [32]. The findings enhance our understanding of the extent to which minority concentrated communities bear a disproportionate burden of environmental pollution. The results presented here provide a starting point for large-scale assessments; identify locations of concern for further investigation; prioritize air toxics and mixtures and emission sources; inform monitoring programs; help communities better understand all sources of environmental risks; and develop more effective targeted risk reduction activities. Although this is a local case study, it is representative of what may be happening in other metropolitan areas in the U.S. and the results could be aggregated to inform national efforts to reduce environmental disparities [33].

Future research should capture multiple attributes of susceptibility and utilize longitudinal designs to give a more comprehensive picture of environmental disparities [33]. The role of racial segregation should be further studied to examine if segregation is more important than other factors in the production of disparities, as Memphis has a high degree of racial residential segregation [34]. Research should also be extended to answer how poor neighborhood characteristics, e.g., substandard housing and poor urban development, may add to the vulnerability of underprivileged populations [35]. Moving disparity research to the indoor environment is also needed, as people typically spend over 70% of their time indoors, and studies have shown elevated levels of multiple pollutants, e.g., lead, particles, allergens, and semi-volatile organic compounds, in the indoor air of minority households [36]. The links between housing and health disparity is more evident and has become the priority of the Environmental Justice Strategy for the U.S. Department of Housing and Urban Development [37].

## 5. Conclusions

Memphis/Shelby County has higher levels of air pollution compared to the state and nation. Air toxics pollution is unevenly distributed in African American concentrated areas. The disproportionate exposure burden is expected to lead to elevated cancer risks and possible adverse health outcomes among minority populations. Population density also modifies the disparity, meaning that densely populated areas may have to deal with higher disease burden and increased medical costs. Risks caused by industrial facilities display a large relative disparity, while on-road mobile sources contribute the largest portion of the total racial disparity. The general public is usually aware that racial disparities in exposure exists, but may not have an accurate sense of the magnitude and sources of disparities. Thus, better risk communication strategies are needed to address the environmental justice issues.

## Appendix

**Table A1:** Groupings of socioeconomic status (SES), racial and population variables identified by principle component analysis. Factor loadings <−0.5 or >0.5 are highlighted.

Variables	Unit	Factor 1	Factor 2
Median household income	(× $1000)	**−0.92**	0.02
Poverty percent	(%)	**0.89**	−0.13
Median house value	(× $1000)	**−0.69**	−0.28
Percent of blacks	(%)	**0.87**	0.17
Percent of females headed house	(%)	**0.94**	0.08
Percent less than high school degree	(%)	**0.92**	−0.16
Total population	(Person)	−0.38	**0.69**
Percent of age >65	(%)	0.12	**−0.63**
Population density	(Person/km^2^)	0.42	**0.50**
